# Light- and Solvent-Responsive Bilayer Hydrogel Actuators with Reversible Bending Behaviors

**DOI:** 10.1021/acsmaterialsau.4c00005

**Published:** 2024-03-22

**Authors:** Gorkem Liman, Esma Mutluturk, Gokhan Demirel

**Affiliations:** † Bio-inspired Materials Research Laboratory (BIMREL), Department of Chemistry, Gazi University, 06500 Ankara, Türkiye; ‡ Department of Chemistry, Polatlı Faculty of Arts and Sciences, Ankara Hacı Bayram Veli University, 06900 Ankara, Türkiye

**Keywords:** Stimuli-responsive polymers, Self-folding, Spiropyran, Smart materials, Hydrogel actuator

## Abstract

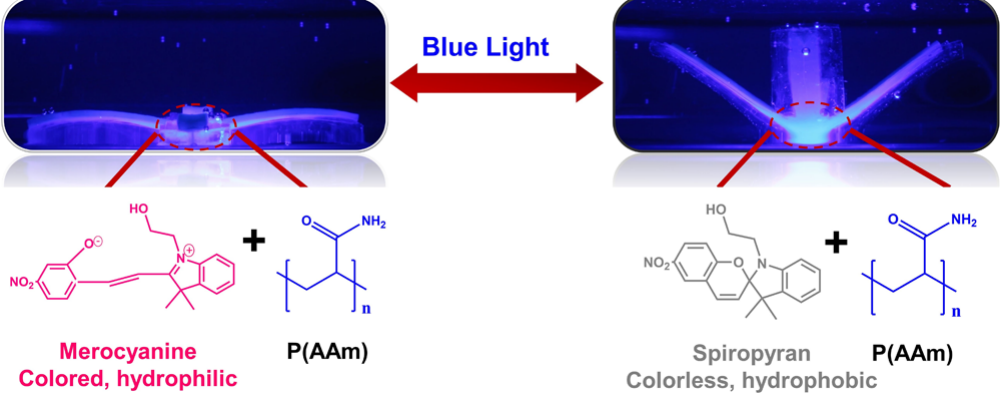

Light-responsive hydrogel systems have gained significant attention due to their unique ability to undergo controlled and reversible swelling behavior in response to light stimuli. Combining light-responsive hydrogels with nonresponsive polymers offers a unique self-folding feature that can be used in soft robotic actuator designs. However, simple formulation of such systems with rapid response time is still a challenging task. Herein, we demonstrate a simple but versatile bilayer polymeric design combining light-responsive spiropyran–polyacrylamide (SP-PAAm) with polyacrylamide (PAAm) hydrogels. The photochromic spiropyran in our polymer design is a closed-ring, hydrophobic compound and turns into an open-ring, hydrophilic merocyanine isomer under light irradiation. The swelling degree of SP-PAAm and PAAm hydrogels was evaluated using LED lights with different wavelengths and solvent media (e.g., water, ethanol, DMF, and DMSO). We observed that SP-PAAm hydrogels reached a swelling ratio of ∼370% with the illumination of the blue LED in the DMF medium. By combining light-responsive SP-PAAm hydrogels with nonresponsive PAAm, a proof-of-concept demonstration was performed to demonstrate the applicability of our fabricated platforms. Although fabricated one-armed bilayer hydrogels possessed self-folding ability with a folding angle of ∼40° in 30 min, the four-armed bilayer platforms demonstrated more efficient and rapid folding behavior and reached a folding angle of ∼75° in ∼15 min. Given their simplicity and efficiency, we believe that such polymeric designs may offer new avenues for the fields of polymeric actuators and soft robotic systems.

## 1 Introduction

**Figure 1:**
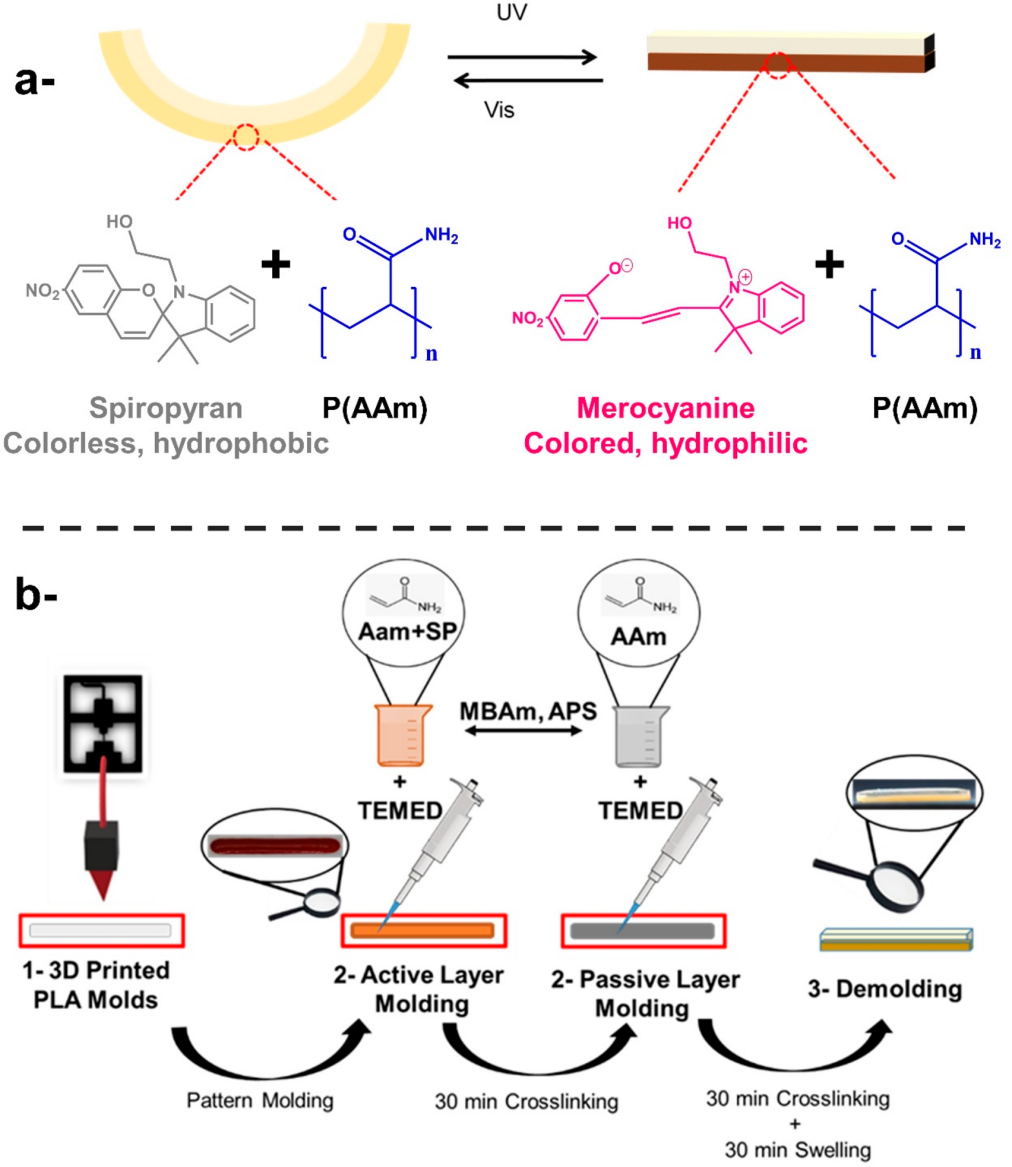
Schematic illustration of the light-responsive SP-PAAm bilayer hydrogel actuators (a). Fabrication of SP-PAAm/PAAm bilayer platforms in one-armed mold (b).

Nature is an inspiration for the design of soft actuators and robots. Robots made of soft materials are gaining popularity due to their ability to morph into different geometries and withstand large deformation.^1−3^ Among the soft materials, hydrogels that can absorb up to 99% of their dry mass in water offer unique possibilities in the fabrication of actuators or robots.^4−7^ The swelling–shrinking process of the hydrogels changes the hydrogel morphology, enabling them to mimic the motion principles found in nature.^8^ Homogeneous expansion–contraction or swelling degree differences in heterogeneous structures can be used to control the swelling behavior of the hydrogel systems, which can be reversibly actuated by light,^9^ temperature,^10^ magnetic field,^11^ or pH.^12^ Particularly, light is an effective external stimulus for hydrogel systems due to its cleanliness and versatility tuned by its wavelength and intensity.^13,14^ To this end, different types of light-responsive hydrogel systems have been developed and used in different fields, including drug delivery, microfluidics, sensing, actuators, robotics, and wearable platforms.^15−22^ Introducing photoresponsive molecules such as spiropyran,^23^ diarylethene,^24^ and azobenzene^25^ to hydrogel systems leads to macroscopic shape changes. Among these molecules, spiropyrans (SPs) that isomerize to protonated merocyanine (MC) under UV light with a wavelength of 330–370 nm and return to the initial colorless state under exposure to visible light (>450 nm) are one of the effective photoresponsive materials (Figure 1a).^26,27^ These two isomers of spiropyran have different chemical structures and physical properties. The SP isomer is a closed ring, uncharged, and nonpolar isomer, whereas the MC isomer is an open ring, zwitterionic, and polar isomer. By taking advantage of these differences in spiropyran structure, several actuator designs have been developed and employed in different applications.^28−30^ For example, Diamond and colleagues reported a bipedal hydrogel walker based on *N*-isopropylacrylamide-*co*-acrylated-spiropyran-*co*-acrylic acid and demonstrated reversible shrinking and swelling via on/off white light irradiation.^31^ In another work, Zhang et al. fabricated a hydrogel actuator as a solvent and temperature-triggered actuator with photoswitchable color-changing behaviors consisting of a poly(*N*-isopropylacrylamide) (PNIPAAm) layer and a PNIPAAm layer with a spiropyran moiety (PNIPAAm-SP).^32^ Considering the recent developments in soft actuator systems, it is still necessary to construct new materials with shape-changing capability under an external stimulus for real-world applications.

In this work, we demonstrate a simple yet versatile strategy to fabricate light- and solvent-triggered bilayer hydrogel actuators with reversible folding capability. Platforms were fabricated within molds with different designs. The hydrogel actuators consist of an active spiropyran–polyacrylamide (SP-PAAm) layer and a passive PAAm part. In the absence of external stimuli, both the active and passive layers swell together in the aqueous medium. The active PAAm layer shrinks rapidly as the light exposure is applied to platforms, whereas the passive PAAm part of the platform tends to keep its volume. As a result of the swelling difference, the platforms self-fold due to the stress created between the layers. Given their simplicity and capability, such stimuli-responsive material designs can offer new possibilities in the field of soft robotics or wearable technologies.

## 2 Experimental Section

### 2.1 Materials

Acrylamide (AAm), *N*,*N*′-methylenebis(acrylamide) (MBAm) 99%, ammonium persulfate (APS), and *N*,*N*,*N*′,*N*′-tetramethylethylenediamine 99.5% (TEMED) were purchased from Sigma-Aldrich (USA) and used as received. 3′,3′-Dimethyl-6-nitro-spiro[2*H*-1-benzopyran-2,2′-indoline]-1′-ethanol (spiropyran, SP) was obtained from Leap Chem Inc. (China). Molds were 3D printed using poly(lactic acid) (PLA) filaments obtained from eSUN (China). Videos of controlled folding were also recorded with a Canon EOS 650D SLR camera (Japan).

### 2.2 Fabrication of Spiropyran–Polyacrylamide/Polyacrylamide Bilayer

The hydrogel systems were fabricated within 3D molds (Figure 1b). To this end, the 3D Builder (Microsoft, USA) and Cura (Ultimaker, Netherlands) software were used to build molds with various patterns (e.g., one- and four-armed). The printing was then carried out with an Ultimaker2+ 3D printer using PLA filaments. A 0.4 mm nozzle diameter, 5% infill density, and 50 mm/s printing speed were employed in the printing process.^9^ Afterward, hydrogel systems with monolayer and bilayer designs were fabricated based on free-radical polymerization using APS as an initiator, TEMED as an accelerator, and MBAm as a cross-linker. The passive layer of the platform was fabricated using AAm, MBAm, and APS. To this end, AAm (1.0 g), MBAm (5.0 mg), and APS (5.0 mg) were dissolved in 5 mL of DMF and purged with nitrogen gas for 10 min. TEMED solution (0.1% v/v, 100 μL) was then added to 350 μL of prepared monomer solution, and the mixture was quickly poured into the molds. For the active SP-PAAm layer, a stock solution containing 1.0 g of AAm and 10 mg of SP in 5 mL of DMF was first prepared. MBAm (5.0 mg) and APS (5.0 mg) were then dissolved into 350 μL of a stock solution prepared previously. After nitrogen purging for 10 min, TEMED (0.1% v/v) was added to this mixture, and the mixture was quickly poured into the pre-cross-linked passive layers. Cross-linking was completed within 30 min at RT in a dark medium. Demolding of cross-linked polymers succeeded after swelling in distilled water for 60 min at RT.

Following the fabrication process, the hydrogel platforms were stimulated with an LED light (470 nm, 50 W) to activate the SP-PAAm active layers in the hydrogel systems and control their folding. For all investigations, the distance between the light source and the platform was kept constant at ∼10 cm, and the illumination angle was 90°.

## 3 Results and Discussion

In our investigation, we fabricated platforms using PAAm and spiropyran. Spiropyran (SP) was chosen as a light-responsive molecule to control polymer folding. The purple-colored merocyanine turns into a cyclic spiropyran upon exposure to visible light. The fabricated platforms were first characterized by FT-IR (Figure S1). The FT-IR spectrum of pure PAAm polymer represents the relatively strong vibration mode at 3335 cm^–1^ corresponding to the symmetrical stretching band of the -NH_2_ group on the PAAm chains, and the shoulder at 3185 cm^–1^ indicates the -NH_2_ stretching in the PAAm for the hydrogen-bonding state. The characteristic peaks at 1648 and 1620 cm^–1^ are also assigned to the C=O group stretching vibration and the N–H bending vibration of amide groups on PAAm chains, respectively. For SP-PAAm, the characteristic peaks for spiropyran were determined in the 750–1600 cm^–1^ spectral region. The bands observed at 1512 and 1570 cm^–1^ correspond to NO_2_ stretching and aromatic ring in spiropyran molecules. FT-IR spectra confirm the fabrication of the platforms. In addition to these, the wettability of the platforms was evaluated using water, DMSO, DMF, and ethanol (Figure S2). To this end, polymeric platforms were first fabricated and dried in a vacuum oven. The fabricated MC-PAAm platforms were also converted into SP-PAAm by exposure to a LED light with a wavelength of 470 nm for 10 min before contact angle measurements. For water, the contact angles were measured as 71.5 ± 1.9° for PAAm, 73.3 ± 1.5° for SP-PAAm, and 67 ± 1.6° for MC-PAAm platforms. As expected, in the presence of SP in the polymer network, the hydrophobicity of the platforms was improved. When SP-PAAm platforms were converted into MC-PAAm, the platforms showed hydrophilic behavior. For other solvents, the contact angle values are tabulated in Table S1.

**Figure 2:**
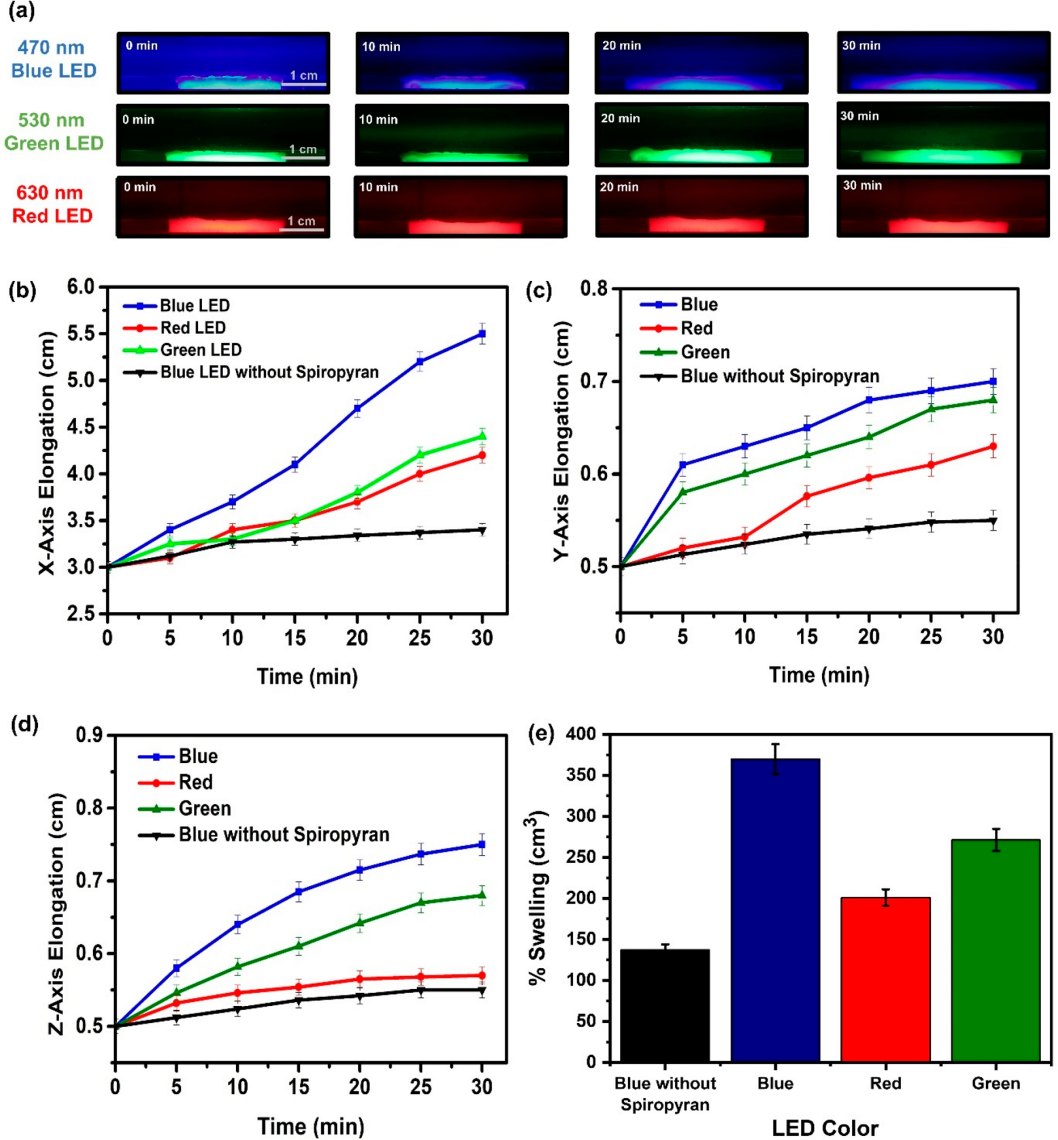
(a) Optical images of the SP-PAAm hydrogels under light illumination with different wavelengths (red (630 nm), blue (470 nm), and green (530 nm)). (b) *X*-axis, (c) *Y*-axis, and (d) *Z*-axis elongations of SP-PAAm and PAAm hydrogels as a function of time. (e) Percent swelling ratios of SP-PAAm and PAAm hydrogels under light illumination with different wavelengths.

To understand the elongation process of our platforms, we compared the swelling behavior of platforms with and without spiropyran. First, spiropyran-containing SP-PAAm hydrogel single layers were fabricated in one-armed molds, and the elongation capabilities of the obtained platforms were investigated under the illumination of LED lights (50 W) with different wavelengths (red (630 nm), blue (470 nm), and green (530 nm)) in DMF medium (Supporting Videos S1–S4). Figure 2a shows the optical images of polymeric platforms under light illumination with different wavelengths at varying time intervals. By analyzing the optical images obtained using IMAGE J software, *X*-axis (length), *Y*-axis (width), and *Z*-axis (thickness) expansions were plotted as a function of time (Figure 2b–d). It was found that the highest expansion in all directions was observed when blue light was used. For the *X*-axis, the elongations for SP-PAAm hydrogel single layers were measured to be ∼1.0 cm for red light, ∼1.25 cm for green light, and ∼2.6 cm for blue light, whereas only 0.25 cm elongation was observed for the platforms without spiropyran under the illumination of blue light. Similar trends of change for the *Y*- and *Z*-axis were also determined for the platforms. In addition to these, the percent swelling of the platforms under light illumination was evaluated, and it was observed that SP-PAAm hydrogels show ∼2.5 times higher swelling performance than the platforms without spiropyran (Figure 2e). It is clear that the elongation performance of the platforms can be manipulated in the presence of light-responsive spiropyran molecules.

**Figure 3:**
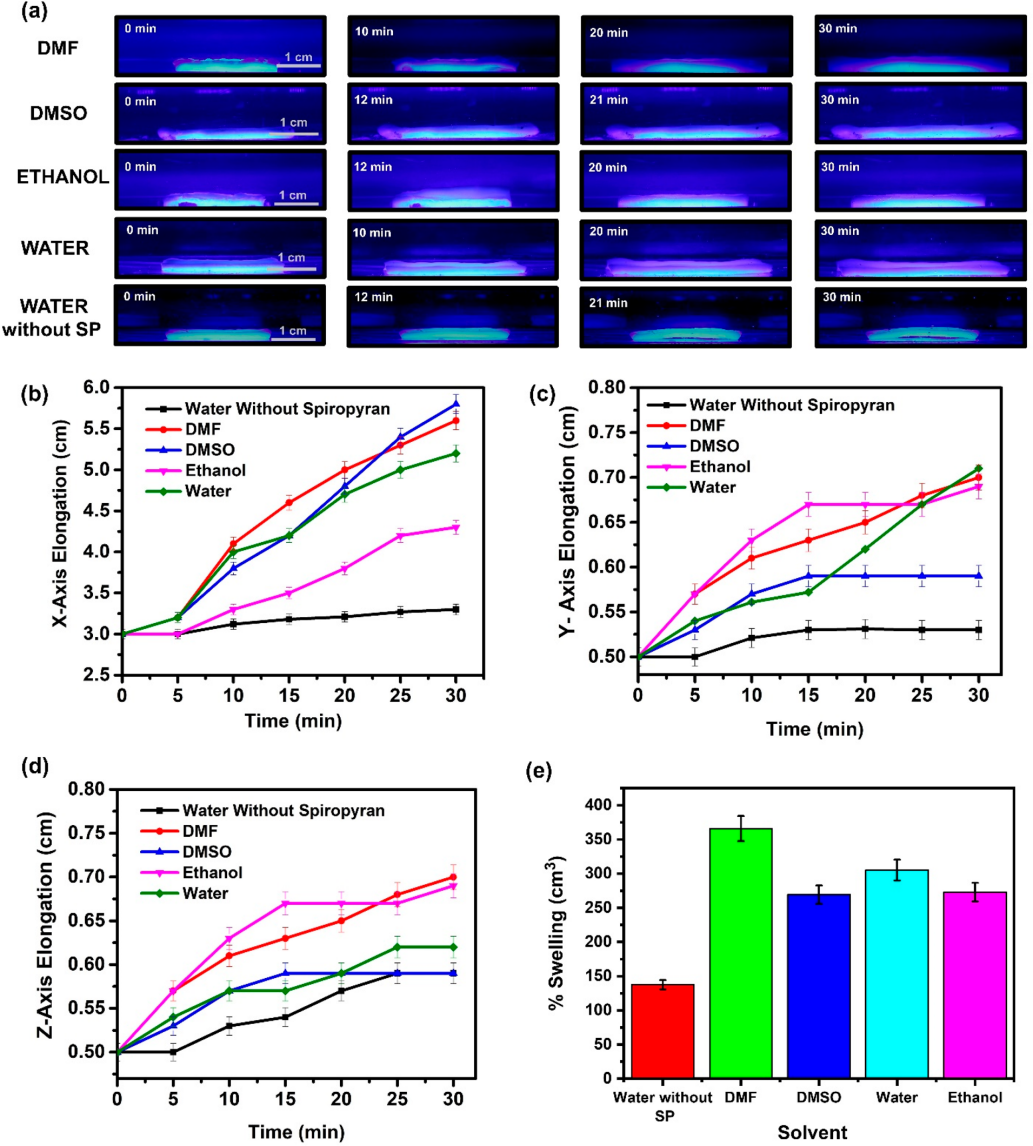
(a) Optical images of the SP-PAAm hydrogels under blue light illumination in DMF, DMSO, ethanol, and water media. (b) *X*-axis, (c) *Y*-axis, and (d) *Z*-axis elongations of SP-PAAm and PAAm hydrogels as a function of time. (e) Percent swelling ratios of SP-PAAm under blue light illumination in different solvent media.

The elongation performance of the SP-PAAm and PAAm hydrogels was also evaluated in different solvents under light illumination (Supporting Video S5–S8). In our investigations, all experiments were performed using blue light, which shows the highest elongations for platforms. Figure 3a illustrates the snapshot optical images of SP-PAAm and PAAm hydrogel platforms in DMF, DMSO, ethanol, and water media under light illumination with a wavelength of 470 nm at varying time intervals. By analyzing the snapshot optical images for platforms, *X*-axis (length), *Y*-axis (width), and *Z*-axis (thickness) expansions were plotted as a function of time (Figure 3b–d). The elongations in the *X*-axis for SP-PAAm hydrogels indicate that they demonstrate the highest elongation in DMSO medium compared to other solvents. The elongations in the *X*-axis were found to be ∼2.7 cm for DMSO, ∼2.5 cm for DMF, ∼2.3 cm for water, and ∼1.1 cm for ethanol, whereas only 0.1 cm elongation was observed for the platforms without spiropyran in water medium. For the *Y*-axis and *Z*-axis, we found quite interesting elongation behavior for platforms. Although DMSO shows the highest elongation in the *X*-axis for platforms with SP, the same hydrogels have lower elongation in the *Y*- and *Z*-axis compared to other solvents. For the *Y*-axis, the highest elongation was observed in water, while the platforms had greater elongation in the *Z*-axis in the DMF solvent medium. This difference is possibly due to the nonhomogeneous distribution of the SP molecules in the polymer network. The overall percent swelling of the platforms was also evaluated, and it was observed that SP-PAAm hydrogels reveal ∼3.0 times higher swelling performance in DMF compared to platforms in water (Figure 3e). It should be noted that the observed elongation results are not based on conventional mass- or volume-based swelling. In our work, we tried to understand the polymer behavior in the presence of SP under light illumination and in different solvent media. Therefore, compared with the conventional swelling behavior of pure PAAm gels, we observed somewhat unexpected results. First of all, it is well-known that pure PAAm hydrogels shrink in DMF, DMSO, and ethanol. In those cases, the free energy for the polymer–polymer contact increases. The polymer chains interact more strongly with each other, and thus, the hydrogel shrinks. During the swelling of heterogeneous hydrogels, the pores inside the network are rapidly filled with the solvent; at the same time, the polymer region takes up solvent from the environment. Thus, two separate processes take place during the swelling of porous networks: (i) solvation of network chains and (ii) filling of the pores by the solvent. In our design, SP molecules play an important role in the elongation of the polymer networks. SP dissolves easily in organic solvents such as DMF, DMSO, and ethanol compared with water. When the SP-PAAm hydrogels are immersed into the solvent media, SP molecules start to dissolve, and much more porous polymer networks form. These pores are then rapidly filled by the solvents, and thus, the polymer platforms elongate in the *X*-, *Y*-, or *Z*-axis. In the absence of SP and light illumination, we only observed shrinkage in these solvents as expected.^33−35^

**Figure 4:**
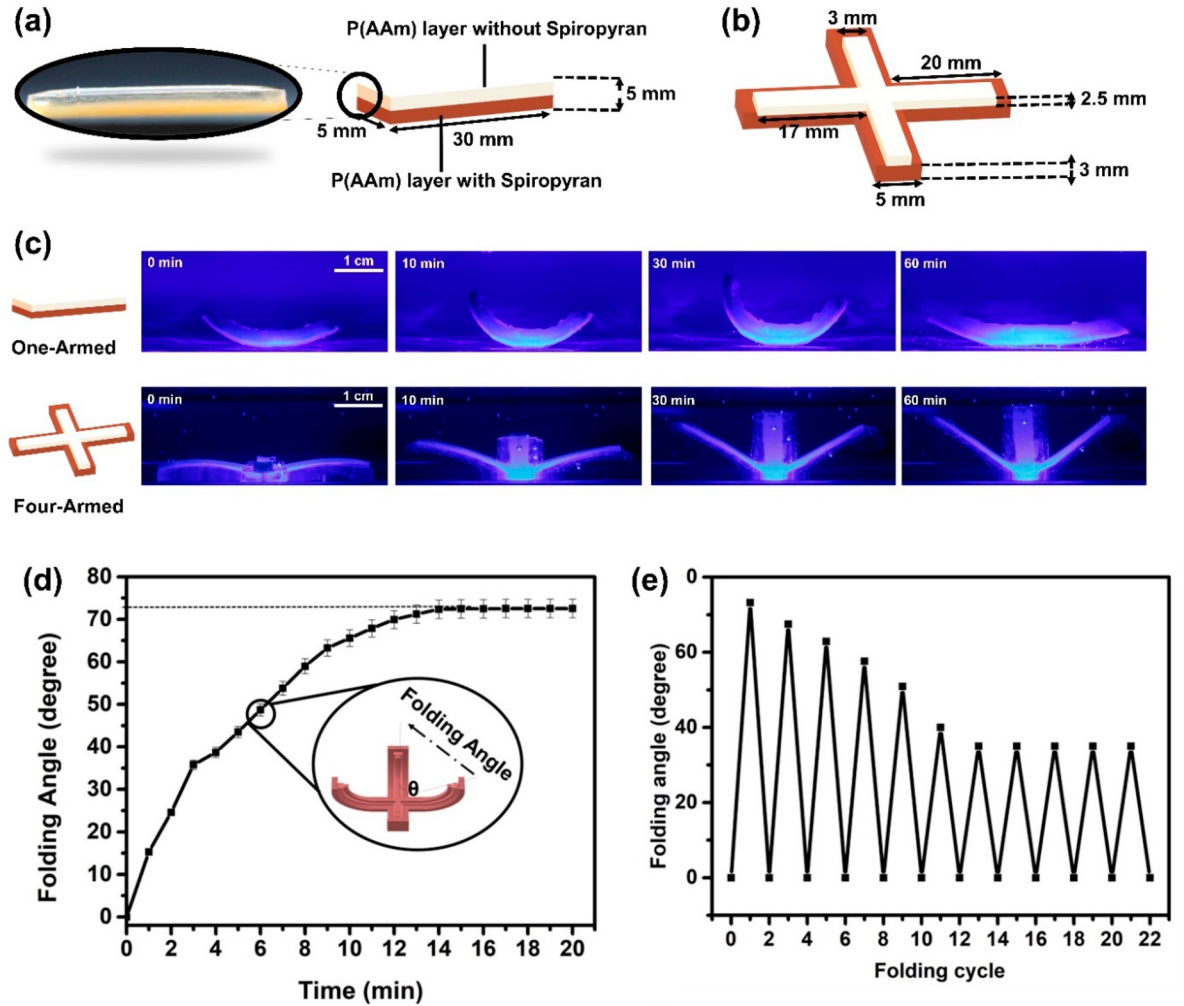
Schematic illustrations for (a) one-armed and (b) four-armed PAAm/SP-PAAm bilayer hydrogels. (c) Optical images of the bilayer hydrogels under blue light illumination in DMF medium. (d) Folding angles for four-armed platforms as a function of time. (e) Effect of reuse of the four-armed platforms on their folding behaviors.

Finally, we performed a proof-of-concept demonstration to show the applicability of our fabricated platforms (Supporting Video S9 and S10). To this end, bilayer hydrogels consisting of nonresponsive PAAm and light-responsive SP-PAAm were fabricated using one- and four-armed molds (Figure 4a,b) as mentioned above. In the design, the light-responsive SP-PAAm hydrogel layer swells in DMF medium, while the nonresponsive PAAm hydrogel layer shrinks. During this process, the swelling difference between the SP-PAAm and PAAm layers creates stress and results in the self-folding of the platform. Figure 4c shows the time-dependent folding process of the bilayer SP-PAAm/PAAm platforms. For the bilayer platform fabricated using one-armed molds, the folding takes place immediately and reaches the highest folding angle of ∼40° in 30 min. However, after 30 min, the folding angle of the platforms decreases, possibly due to the release of the SP molecules from the bilayer. Similar to one-armed platforms, the four-armed bilayers demonstrate an effective folding behavior and reach a folding angle of ∼75° in ∼15 min (Figure 4d). The repeatability of the folding process for the four-armed platforms was also evaluated (Please also see Figure S3 for the folding/defolding process). To this end, the platforms first folded in the presence of blue light. After 30 min, the folded platform was exposed to UV light for 24 h to transform SP molecules to MC in the bilayer structure. In each cycle, as demonstrated in Figure 4e, the platforms underperform in their folding ability because the light-responsive molecules in the network structure release from the platforms.

## 4 Conclusions

In this work, we demonstrated light/solvent responsive hydrogel bilayers based on spiropyran–polyacrylamide (SP-PAAm) and polyacrylamide (PAAm) hydrogels. The fabricated bilayer hydrogels were folded in a controlled manner in the presence of light illumination. The swelling degree of SP-PAAm and PAAm hydrogels were evaluated using LED lights with different wavelengths and solvent media (e.g., water, ethanol, DMF, and DMSO). We found that SP-PAAm hydrogels reach a swelling ratio of ∼370% with the illumination from blue LEDs in DMF medium. Combining light-responsive SP-PAAm hydrogels with nonresponsive PAAm, we performed a proof-of-concept demonstration to show the applicability of our fabricated platforms. Although one-armed bilayer hydrogels show self-folding with an angle of ∼40° in 30 min, four-armed bilayer platforms demonstrate more efficient folding behavior and reach a folding angle of ∼75° in only ∼15 min. Such hydrogel systems could be combined with different polymeric or inorganic materials that have the ability to respond to magnetic fields, light, or pH for the development of effective soft robotic systems. Moreover, by combining 3D printing or two-photon lithography techniques, we could apply our design strategy for varying biomedical applications. We believe that the polymeric design reported herein may open up exciting new possibilities for the development of soft robotic platforms and find a place in real-life applications.

## Supporting Information Available

FT-IR spectra of the fabricated platforms, contact angle measurements of the platforms, and comparison between this work and existing SP based polymeric systems (PDF)
Swelling of SP-PAAm platforms in the presence of blue light illumination (MP4)
Swelling of SP-PAAm platforms in the presence of red light illumination (MP4)
Swelling of SP-PAAm platforms in the presence of green light illumination (MP4)
Swelling of PAAm platforms in the presence of blue light illumination (MP4)
Swelling of SP-PAAm platforms in DMF medium (MP4)
Swelling of SP-PAAm platforms in DMSO medium (MP4)
Swelling of SP-PAAm platforms in ethanol medium (MP4)
Swelling of SP-PAAm platforms in water medium (MP4)
Folding of one-armed SP-PAAm/PAAm platforms (MP4)
Folding of four-armed SP-PAAm/PAAm platforms (MP4)
