# Supplementary material for: Light- and Solvent-Responsive Bilayer Hydrogel Actuators with Reversible Bending Behaviors
Source: ACS Mater Au. 2024 Mar 22;4(4):385–92. doi: 10.1021/acsmaterialsau.4c00005 (PMC11240406; doi:10.1021/acsmaterialsau.4c00005)
Supplement: Supplementary file 1 — mg4c00005_si_001.pdf [file mg4c00005_si_001.pdf]

Supporting Information

Light- and Solvent-Responsive Bilayer Hydrogel Actuators with Reversible Bending Behaviors

Gorkem Liman[1], Esma Mutluturk[2*] and Gokhan Demirel[1*]

[1]Bio-inspired Materials Research Laboratory (BIMREL), Department of Chemistry, Gazi University, 06500 Ankara, Türkiye
[2]Department of Chemistry, Polatlı Faculty of Arts and Sciences, Ankara Hacı Bayram Veli University, 06900, Ankara, Türkiye

The PDF file includes:

Figure S1. FT-IR spectra of Spiropyran (a), SP-P(AAm) (b), and pure P(AAm) (c).

Figure S2. Optical images of water droplets on SP-P(AAm), P(AAm), and MC-P(AAm) platforms.

Figure S3. Snapshot images of the folding and de-folding process of SP-P(AAm)/ P(AAm) bilayer hydrogel platforms.

Table S1. Contact angle values of SP-P(AAm), P(AAm), and MC-P(AAm) using different solvents.

Table S2. Comparison between this work and existing SP-based polymeric systems.

Other Supplementary Materials for this manuscript include the following:

Video S1 (.mp4 format) Swelling of SP-P(AAm) platforms in the presence of Blue Light illumination.

Video S2 (.mp4 format) Swelling of SP-P(AAm) platforms in the presence of Red Light illumination.

Video S3 (.mp4 format) Swelling of SP-P(AAm) platforms in the presence of Green Light illumination.

Video S4 (.mp4 format) Swelling of P(AAm) platforms in the presence of Blue Light illumination.

Video S5 (.mp4 format) Swelling of SP-P(AAm) platforms in DMF medium.

Video S6 (.mp4 format) Swelling of SP-P(AAm) platforms in DMSO medium.

Video S7 (.mp4 format) Swelling of SP-P(AAm) platforms in Ethanol medium.

Video S8 (.mp4 format) Swelling of SP-P(AAm) platforms in Water medium.

Video S9 (.mp4 format) Folding of one-armed SP-P(AAm)/P(AAm) platforms.

Video S10 (.mp4 format) Folding of four-armed SP-P(AAm)/P(AAm) platforms.

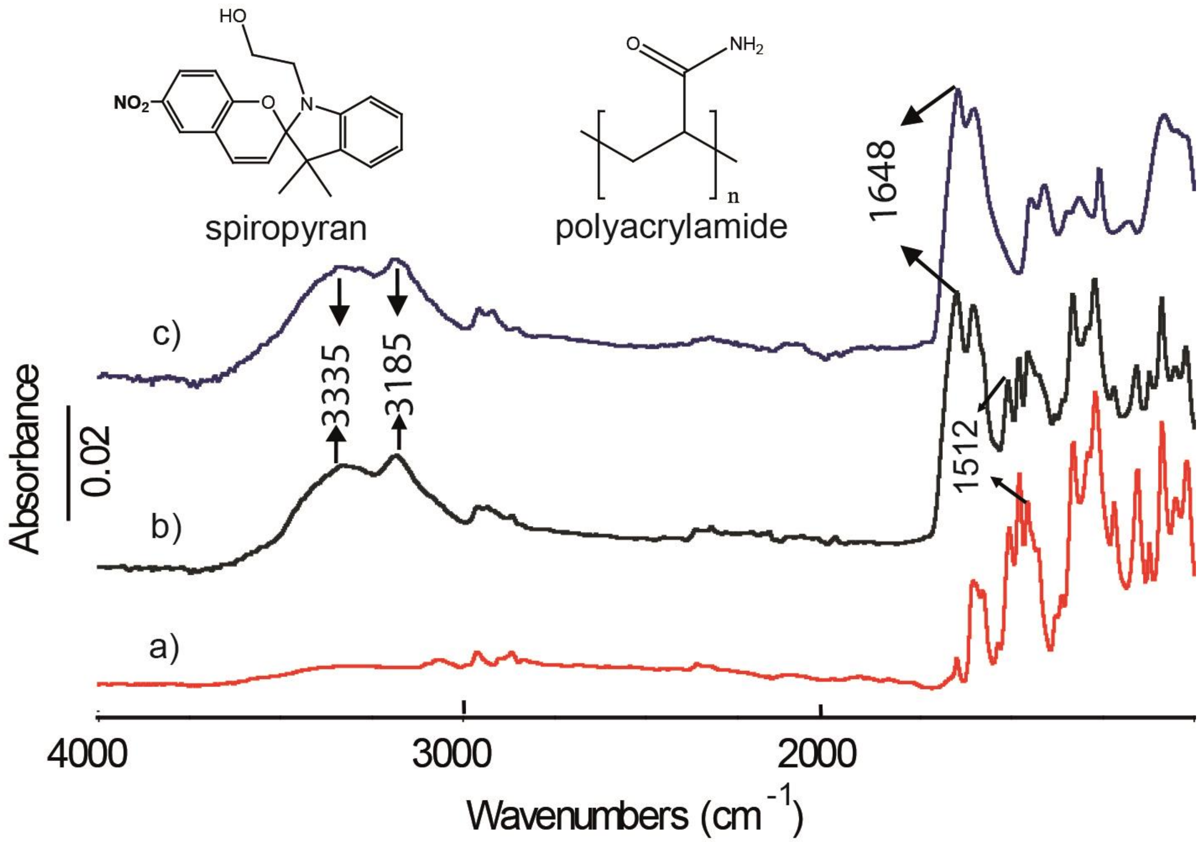

Figure S1. FT-IR spectra of Spiropyran (a), SP-P(AAm) (b), and pure P(AAm) (c).

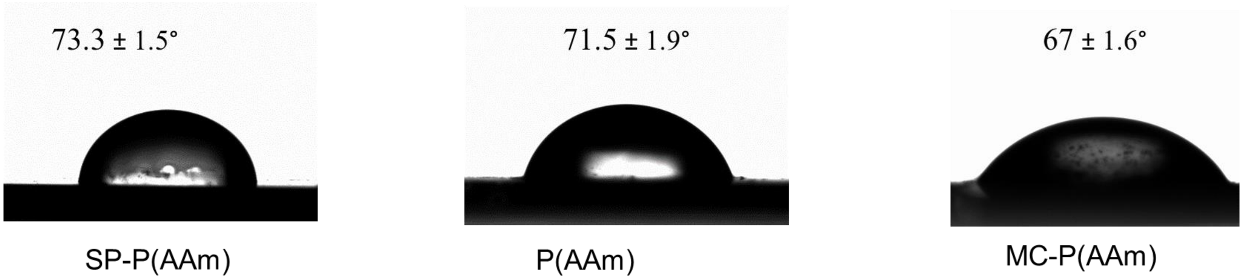

Figure S2. Optical images of water droplets on SP-P(AAm), P(AAm), and MC-P(AAm) platforms.

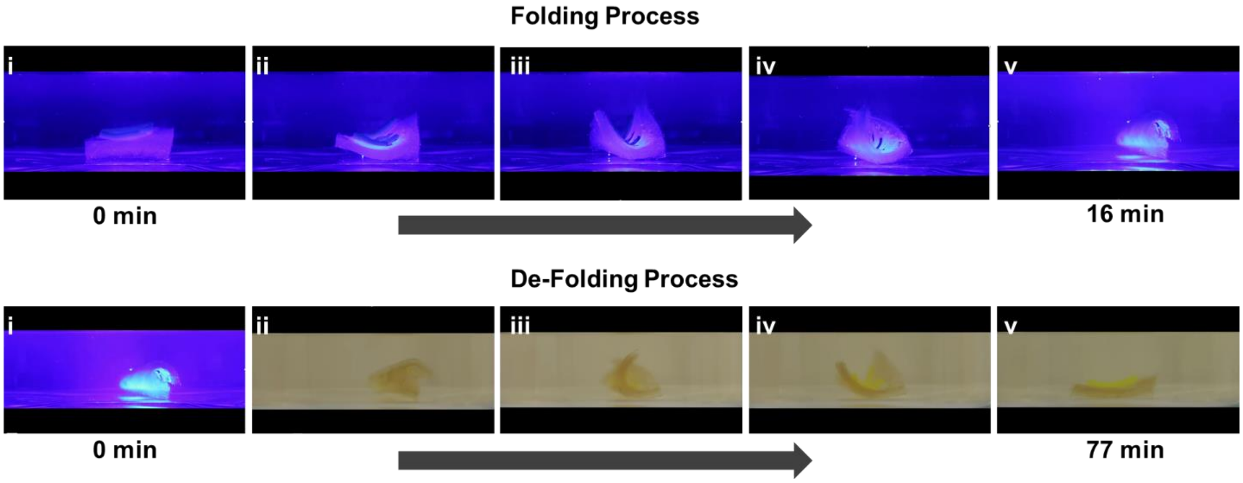

Figure S3. Snapshot images of the folding and de-folding process of SP-P(AAm)/P(AAm) bilayer hydrogel platforms as a function of time.

Table S1. Contact angle values of SP-P(AAm), P(AAm), and MC-P(AAm) using different solvents.

Solvent	SP-PAAm	P(AAm)	MC-P(AAm)
DMSO	75.3 ± 2.4°	73.4 ± 1.7°	66 ± 2.3°
DMF	80.1 ± 1.3°	72.1 ± 2.6°	64 ± 1.8°
ETHANOL	96.2 ± 2.5°	93.5 ± 1.2°	77 ± 2.1°
WATER	73.3 ± 1.5°	71.5 ± 1.9°	67 ± 1.6°

Table S2. Comparison between this work and existing SP based polymeric systems.

System	Light Source	Response Time	Application	Reference
P(NIPAM-*co*-MA-*co*-SPMA) and P(NIPAM-*co*-MA) bilayer	UV	30 min	Bending	(1)
N- p(NIPAAm-*co*-SP-*co*-AA) hydrogel	Vis	21min	Hydrogel Walker	(2)
SP doped polyethylmethacrylate-*co*-methylacrylate polymer film	He–Ne Laser	12 second	Switching and Bending Polymer Film	(3)
P(AAm-co-VDT-co-SPAA)	UV light	15 min	Gene Delivery	(4)
PNIPAM and P(AAm-SP) bilayer	Blue LED (450 nm)	15 second	Origami Folding	(5)
P(AAm-SP-Azo) hydrogels	Blue LED (440 nm)	20 min	Stimuli-Responsive Hydrogels	(6)
p(AAm-*co*-AAc) hydrogel	IR Laser	20 min	Self Trapped Beams	(7)
P(AAm-SP-Azo) hydrogel	Blue LED (440 nm)	100 sec	Self Healable Polymer	(8)
This Work	Blue LED (440 nm)	15 min	Folding	-

Abbreviations

Poly (isopropyl acrylamide): NIPAAm

Poly(acrylic acid):PAA

Methyl acrylate: MA

Azobenzene: Azo

2-vinyl-4,6-diamino-1,3,5-triazine: VDT

Spiropyran: SP

REFERENCES

1. Long, S.; Huang, J.; Xiong, J.; Liu, C.; Chen, F.; Shen, J.; Huang, Y.; Li, X. Designing Multistimuli-Responsive Anisotropic Bilayer Hydrogel Actuators by Integrating LCST Phase Transition and Photochromic Isomerization. *Polymers* **2023**, *15*, 786. doi.org/10.3390/polym15030786.
2. Francis, W.; Dunne, A.; Delaney, C.; Florea, L.; Diamond, D. Spiropyran Based Hydrogels Actuators─Walking in the Light. *Sens. Actuators, B.* **2017**, 250, 608– 616, DOI: 10.1016/j.snb.2017.05.005.
3. Athanassiou, A.; Lakiotaki, K.; Kalyva, M.; Georgiou, S.; Fotakis, C. Photoswitches Operating upon Ns Pulsed Laser Irradiation. *Appl. Surf. Sci.* **2005**, *248*, 56– 61, DOI: 10.1016/j.apsusc.2005.03.077.
4. Wang, N.; Li, Y.; Zhang, Y.; Liao, Y.; Liu, W. High-Strength Photoresponsive Hydrogels Enable Surface-Mediated Gene Delivery and Light-Induced Reversible Cell Adhesion/Detachment. *Langmuir* **2014**, *30*, 11823– 11832, DOI: 10.1021/la502916j.
5. Li, C.; Xue, Y.; Han, M.; Palmer, L. C.; Rogers, J. A.; Huang, Y.; Stupp, S. I. Synergistic Photoactuation of Bilayered Spiropyran Hydrogels for Predictable Origami-Like Shape Change. *Matter* **2021**, *4*, 1377– 1390, DOI: 10.1016/j.matt.2021.01.016
6. Liu, A.; Xiong, C.; Ma, X.; Ma, W.; Sun, R. A Multiresponsive Hydrophobic Associating Hydrogel Based on Azobenzene and Spiropyran. *Chin. J. Chem.* **2019**, **37**, 793–798.
7. Meeks, A.; Mac, R.; Chathanat, S.; Aizenberg, J. Tunable long-range interactions between self-trapped beams driven by the thermal response of photoresponsive hydrogels. *Chem.Mater.* **2020**, *32*, 10594– 10600, DOI: 10.1021/acs.chemmater.0c03702.
8. Xiong, C.; Zhang, L.; Xie, M.; Sun, R. Photoregulating of Stretchability and Toughness of a Self-Healable Polymer Hydrogel. *Macromol. Rapid Commun.* **2018**, *39*, 1800018 DOI: 10.1002/marc.201800018.
